# Case report: Sclerosed hemangioma of the liver: A diagnostic challenge

**DOI:** 10.3389/fsurg.2022.985849

**Published:** 2022-12-29

**Authors:** M. Poras, G. Katsanos, A. C. Agrafiotis, P. Demetter, M. Pezzullo, V. Lucidi

**Affiliations:** ^1^ Department of Abdominal Surgery, St Pierre University Hospital (Université Libre de Bruxelles), Brussels, Belgium; ^2^ Department of Abdominal Surgery, Erasme University Hospital (Université Libre de Bruxelles), Brussels, Belgium; ^3^ Department of Pathology, Erasme University Hospital (Université Libre de Bruxelles), Brussels, Belgium; ^4^ Department of Radiology, Erasme University Hospital (Université Libre de Bruxelles), Brussels, Belgium

**Keywords:** sclerosed, sclerosing, hemangioma, cholangiocarcinoma, liver

## Abstract

Hemangiomas are the most common noncystic benign hepatic tumors and are usually incidentally discovered during routine radiological examinations. The diagnosis of hepatic hemangiomas with a typical presentation is generally easy with plain and cross-sectional imaging; however, it can be complicated when hemangiomas undergo histological changes such as fibrosis. Sclerosed hepatic hemangioma (SHH) is the extreme presentation of this fibrotic process. These atypical lesions can be misdiagnosed as primary hepatic malignancies or metastasis. Their diagnosis is established by histological examination. We report the case of a patient with an SHH, which was misdiagnosed as an intrahepatic cholangiocarcinoma. This article's aim is to draw attention to this infrequent pathology and underline the features of this benign tumor that could suggest its diagnosis prior to surgery to avoid unnecessary hepatic resections.

## Introduction

Hemangioma is the most common noncystic benign hepatic tumor with an incidence of 1%–20% in autopsy studies (1, 2). In a case series including 2008 patients who underwent hepatic resection, hemangiomas accounted for 41.7% of benign tumors (3). They present a female predilection around 30–50 years old (4, 5). In their vast majority, they are asymptomatic, and they are usually an incidental finding during routine radiological examinations or during laparotomy or laparoscopy for other abdominal pathologies (5, 6). When symptomatic, the main manifestation is abdominal pain or discomfort, and the prevalence of complications (pain, enlarging mass, rupture, Kasabach–Merritt syndrome) is extremely low (5, 7). Occasionally, tumor enlargement is possible during pregnancy or treatment with oral contraception (4). Spontaneous regression of hemangiomas occurs rarely (8).

Typical hemangiomas can be safely diagnosed during an ordinary radiological workup with ultrasonography (US), computed tomography (CT), and/or magnetic resonance imaging (MRI) (9). However, the diagnosis of hemangiomas can be challenging when the lesions are complicated with necrosis, fibrosis, or calcification (10). Sclerosed hepatic hemangioma (SHH) is a rare entity, with only 78 cases described in the literature, including this report. Approximately 70% of SHH are diagnosed by surgical resection vs. 25% by biopsy or radiology (11).

In the case reported herein, an SHH was misdiagnosed as an intrahepatic cholangiocarcinoma, leading to surgical resection. The tumor presented atypical features on radiological examinations, and a definitive diagnosis was established on histological examination.

## Case report

We report the case of an 85-year-old Caucasian female patient who presented at the outpatient clinic of gastroenterology with the recent onset of vague abdominal pain localized in the epigastrium and the right subcostal area. There were no signs of jaundice. Clinical examination was normal.

Relevant clinical history included a right hemicolectomy and adjuvant chemotherapy for an adenocarcinoma of the colon, 16 years ago.

Laboratory tests were within normal limits.

Tumor markers such as carcinoembryonic antigen (CEA), alpha fetoprotein (a-fp), and CA 19-9 were within normal limits. Gastroscopy and colonoscopy did not detect any lesions. An abdominal US showed a hypoechogenic lesion at the level of the segment IV of the liver. On CT, the lesion showed no enhancement in the arterial phase and no dynamic changes between the portal venous phase and the delayed phase, showing weak and heterogeneous mainly peripheral enhancement. An abdominal MRI showed a mass (3 cm on its greatest diameter) with malignant features in segments III and IV. There was no intrahepatic biliary obstruction. A contrast-enhanced ultrasound (CEUS) was performed a few days after MRI confirmed the absence of any centripetal enhancement.

There were no previous examinations available for comparison in our archives.

**Figure 1:**
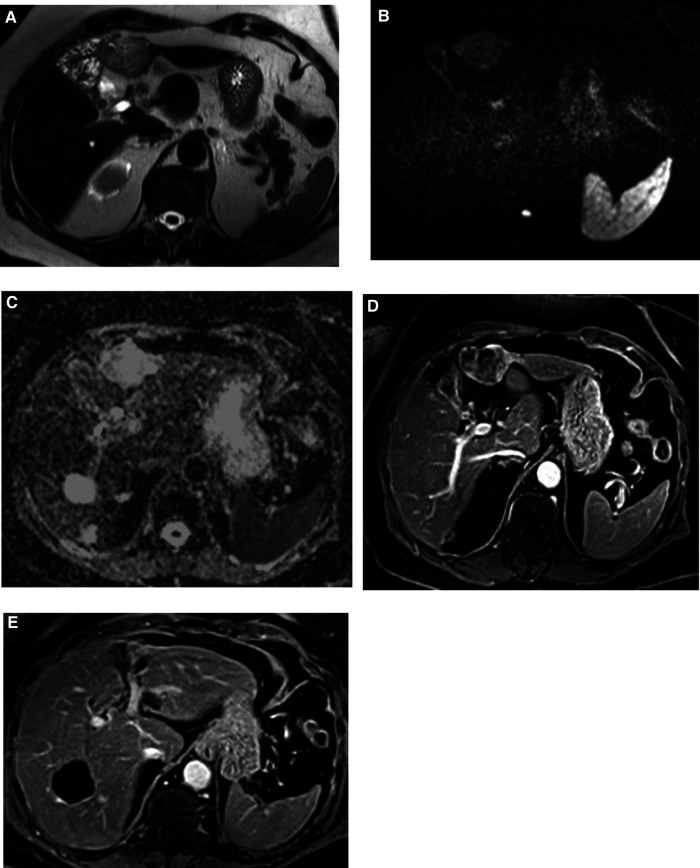
MRI images. Focal oval lesion straddling segments III and IVb showing sharp contours, heterogeneously high signal in T2 (**A**) and in diffusion-weighted imaging (**B**) corresponding to heterogeneous hypersignal on ADC map (**C**). Irregular and globally hypoenhancing behavior on T1 portal venous phase (**D**). (**E**) The lesion was located inferiorly to segmental left portal bifurcation, displaying a contact with the segmental branch for segment III without any major distortion or infiltration.

The overall appearance was highly suggestive of an intrahepatic cholangiocarcinoma (Figure 1).

A fluorodeoxyglucose (FDG) positron emission tomography/CT (PET/CT) scan did not show any high FDG uptake in the liver or elsewhere. Despite a negative PET scan, with the rest of the imaging studies suggesting cholangiocarcinoma, the decision of a multidisciplinary reunion was in favor of a surgical resection.

During surgery, a white-colored, well-demarcated soft tumor was identified at the junction of segments III and IV. There were no enlarged lymph nodes in the hepatoduodenal ligament, and there were no signs of peritoneal carcinomatosis. Perioperative liver ultrasound did not detect other lesions.

**Figure 2:**
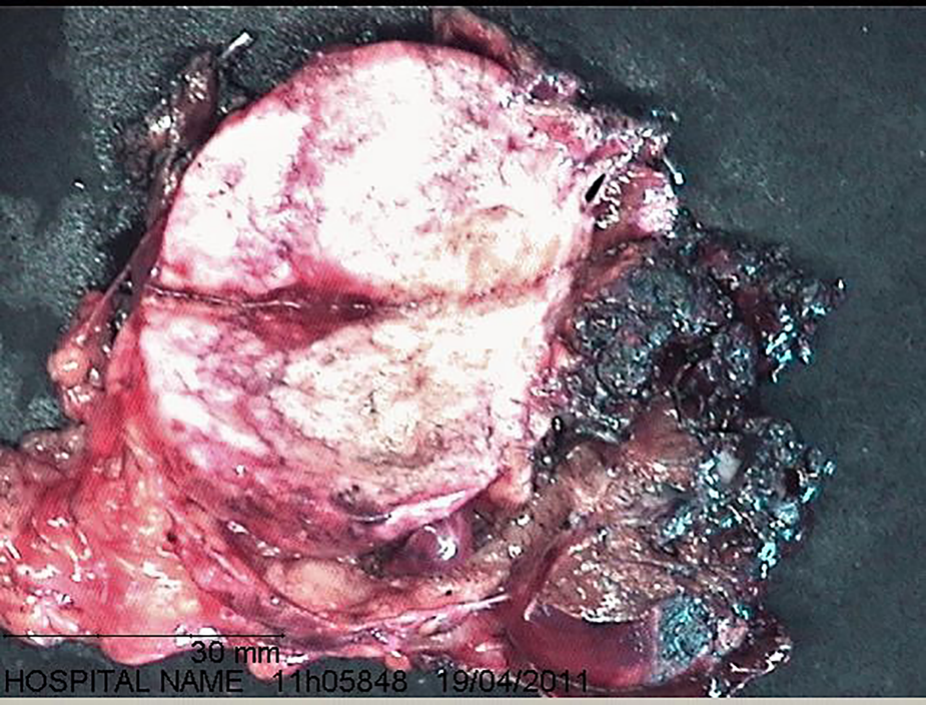
Predominantly extrahepatic indurated tumoral mass.

During resection, the tumor was found to be in contact with the left hepatic duct, which was confirmed by a perioperative cholangiography and a left hepatectomy was performed (Figure 2). Intraoperative histological analysis was not performed.

The postoperative course was uneventful, and the patient was discharged on the seventh postoperative day.

**Figure 3:**
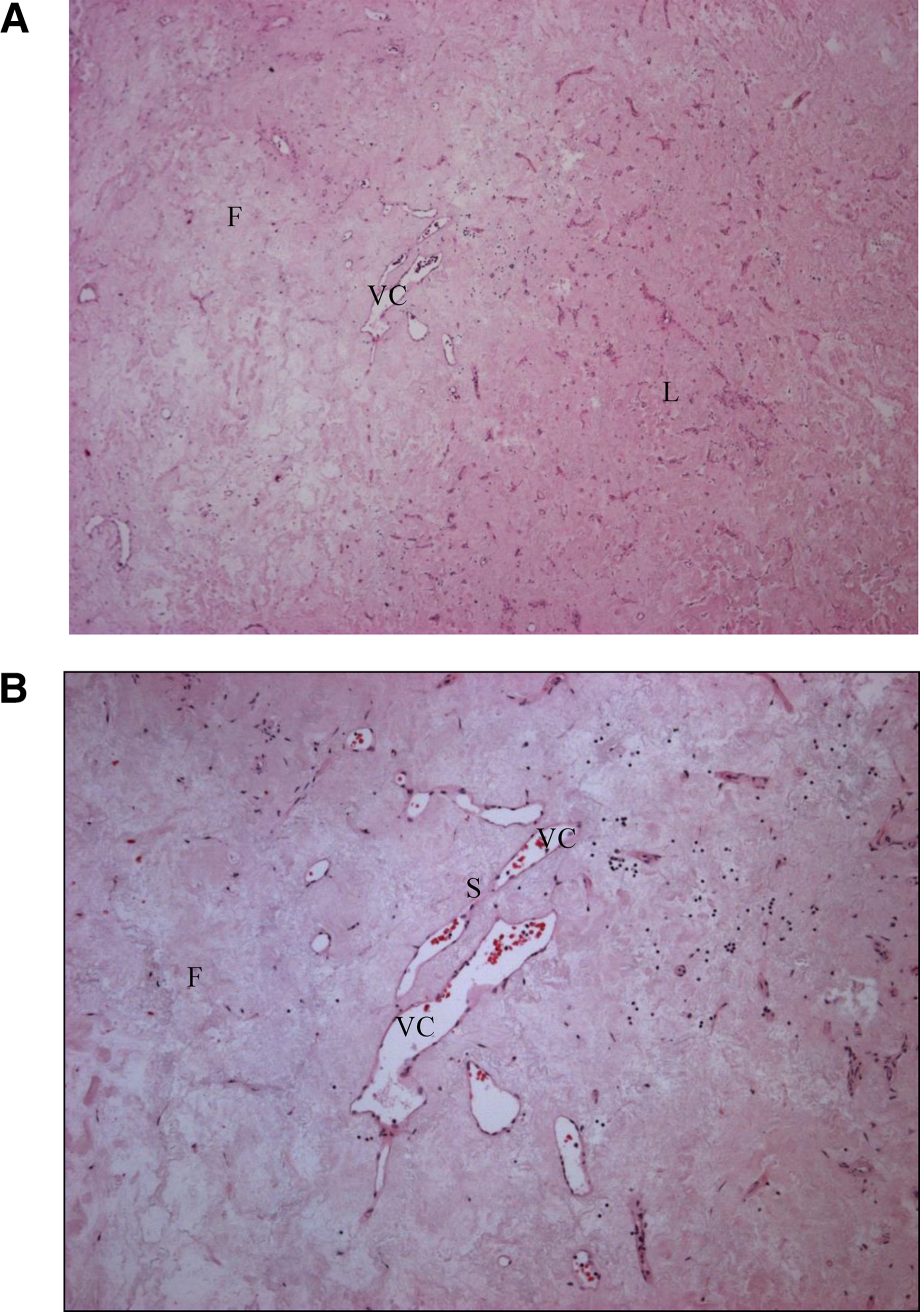
Sclerosed hemangioma, histological appearances. A rich paucicellular fibrous (F) stroma is distinguished from the Normal liver (L) tissue. Irregular vascular channels (VC) are separated by endothelial cells with thin fibrous septa (S). (**A**) Histology. Hematoxylin–eosin, magnification ×40. (**B**) Histology, Hematoxylin–eosin, magnification ×100.

Microscopic examination showed a fibrous stroma and the presence of vascular structures, with no malignant features (Figures 3A,B). The histological image was compatible with a sclerosed hemangioma.

## Discussion

SHH is an infrequent variant of hemangioma and is exceedingly difficult to differentiate from hepatic malignancies (11). There is an intense presence of fibrous tissue in which small vessels are occasionally detected (12).

Typical hemangiomas can be safely diagnosed during an ordinary radiological workup with US and CT/MRI (9). Hemangiomas present as hyperechoic, homogeneous lesions compared with normal parenchyma on US (4). Nevertheless, large or massive hemangiomas may also contain heterogeneous areas (7). On unenhanced CT scan, hemangiomas present as hypodense areas similar to liver vessels. After contrast injection, there is peripheral nodular enhancement and a fill-in of the lesion over time (5). Hemangiomas present with a hypointense signal on T1 IRM and a strongly hyperintense signal on T2-weighted images. The dynamic behavior with centripetal progressive enhancement is the same as described for CT (4, 5, 13, 14). MRI is the best performing imaging modality to diagnose liver hemangiomas with high specificity and sensitivity rates (4, 5, 14). Arteriography is rarely used prior to surgery (7). There is no uptake on PET scan (4). In a series of hepatic masses, including two typical hemangiomas, these lesions had no increased uptake on 18F-FDG PET, and on the other hand, they presented as hypometabolic regions on 11C-acetate PET imaging (15).

On the contrary, as stressed by Yamashita et al. in the case of SHH, the interpretation of radiological features alone can often lead to misdiagnosis, as they are similar to those of hepatic malignancies (2, 16, 17). In the radiological study by Jia et al. in 2021, 75% of SHH were misdiagnosed (18). With fewer than 80 cases of SHH found in the literature, it is a challenge to diagnose them preoperatively (11).

Doyle et al. in their retrospective study of 10 histologically proven SHH, found imaging features suggestive of the lesion, which, however, do not permit a definitive diagnosis. These features include a geographic pattern, capsular retraction, a decrease in size over time, and the loss of previously enhanced areas (1).

Mori et al. analyzed the imaging characteristics of 11 SHH, and when US was available, the lesions were hyperechoic (2).

On plain CT, SHH often presented a low density with irregular shape and heterogeneous density in the majority of cases (18). After contrast injection, the majority of SHH had atypical enhancement characteristics, with little enhancement, or no enhancement during the arterial phase (2, 18). This atypical enhancement pattern could be related to the degree of degeneration with the obliteration of vascular channels and extensive tissue fibrosis (18).

The apparent diffusion coefficient (ADC) can be helpful in the differentiation between SHH and hepatic malignant tumors, as in SHH the ADC values are higher than the surrounding liver parenchyma, suggesting a benign lesion (18, 19). However, an ADC threshold value definition is not defined, because of the great individual variability (18). We do not receive help from this coefficient for the patient.

So, even with recent developments in radiological modalities, imaging alone cannot establish a definitive diagnosis. Small size or fibrosis can further accentuate the diagnostic challenge, as the amount of fibrosis in a sclerosing hemangioma figures out its morphological characteristics and the dynamic behavior that can shift progressively from the classical centripetal filling of typical hemangiomas to the weak and progressive enhancement of fibrotic tissue.

Fine needle aspiration or core biopsy procedures are generally safe with a low incidence of hemorrhagic complications especially in the case of SHH as it is less vascular than cavernous hemangioma (4, 12). Percutaneous fine needle biopsy should be the procedure of choice to distinguish degenerated hemangioma from hepatocellular carcinoma as advocated by Cheng et al. (20). CT-guided biopsy may be useful and can avoid major surgery (21). On the other hand, the risk of rupture or seeding in case of malignancy during biopsy should be taken into account in the decision-making process (2, 22). The above, along with the rarity of sclerosing hemangioma and the proposed diagnosis of cholangiocarcinoma, was the reason that we did not perform a preoperative biopsy in the present case.

At microscopic examination, cavernous hemangiomas present as vascular channels of different sizes with flattened endothelial cells separated by connective tissue septa (10). In fact, when partial fibrosis occurs, they are called sclerosing hemangiomas and when vascular spaces are extensively occupied by fibrous tissue, they are called sclerosed cavernous hemangiomas (2, 23). Makhlouf and Ishak describe the features of sclerosing and sclerosed hemangiomas and underline the role of mast cells in the pathogenesis of these variants of hepatic hemangiomas (23). The flattened cells show positivity for the endothelial marker, factor VIII-related antigen, marking the vascular origin of this tumor. This immunohistochemical staining is of paramount importance in differentiating SHH from malignant hepatic tumors, primary or metastatic (12).

Surgical resection is reserved for symptomatic patients, in cases where imaging techniques and histological examination after percutaneous biopsies are not helpful, and in cases with a high suspicion for malignancy due to medical history. In the other cases, a simple observation is sufficient.

## Conclusion

SHH is an extremely rare benign tumor, and it is a challenge to differentiate from hepatic malignant tumors. SHH has an excellent prognosis and can be followed without surgery. Imaging interpretation alone can lead to a misdiagnosis; however, there are features that could raise the suspicion of an SHH. In that case, a preoperative biopsy or perioperative frozen section is important to avoid unnecessary hepatic resections. If neither imaging interpretation nor biopsy can establish a diagnosis, or if biopsy is contraindicated, the least invasive resection should be performed.

## Data Availability

The original contributions presented in the study are included in the article/Supplementary Material, further inquiries can be directed to the corresponding author.
